# “Giving us hope”: Parent and neonatal staff views and expectations of a planned family‐centred discharge process (Train‐to‐Home)

**DOI:** 10.1111/hex.12514

**Published:** 2016-12-21

**Authors:** Jenny Ingram, Maggie Redshaw, Sarah Manns, Lucy Beasant, Debbie Johnson, Peter Fleming, David Pontin

**Affiliations:** ^1^ University of Bristol Bristol UK; ^2^ University of Oxford Oxford UK; ^3^ University of the West of England Bristol UK; ^4^ University of South Wales Pontypridd UK

**Keywords:** discharge planning, family‐centred, neonatal care, parental self‐efficacy

## Abstract

**Background:**

Preparing families and preterm infants for discharge is relatively unstructured in many UK neonatal units (NNUs). Family‐centred neonatal care and discharge planning are recommended but variable.

**Design and participants:**

Qualitative interviews with 37 parents of infants in NNUs, and 18 nursing staff and 5 neonatal consultants explored their views of discharge planning and perceptions of a planned family‐centred discharge process (Train‐to‐Home). Train‐to‐Home facilitates communication between staff and parents throughout the neonatal stay, using a laminated train and parent booklets.

**Results:**

Parents were overwhelmingly positive about Train‐to‐Home. They described being given hope, feeling in control and having something visual to show their baby's progress. They reported positive involvement of fathers and families, how predicted discharge dates helped them prepare for home and ways staff engaged with Train‐to‐Home when communicating with them. Nursing staff reactions were mixed—some were uncertain about when to use it, but found the visual images powerful. Medical staff in all NNUs were positive about the intervention recognizing that it helped in communicating better with parents.

**Conclusions:**

Using a parent‐centred approach to communication and informing parents about the needs and progress of their preterm infant in hospital is welcomed by parents and many staff. This approach meets the recommended prioritization of family‐centred care for such families. Predicted discharge dates helped parents prepare for home, and the ways staff engaged with Train‐to‐Home when communicating with them helped them feel more confident as well as having something visual to show their baby's progress.

## 1 Background

Parents of preterm infants need to learn how to look after their infants after discharge and to prepare themselves and their home. Preparation for discharge is relatively unstructured in many UK neonatal units (NNUs) and may be left until late in the hospital stay.^1^, ^2^, ^3^ This is linked to staff telling parents that babies will go home around their original expected date of delivery (EDD), despite increasing evidence of shorter hospital stays and earlier discharge home due to neonatal care improvements. Discharging babies before EDD commonly leaves parents feeling unprepared and lacking confidence.^1^, ^2^, ^3^

### 1.1 The importance of involving parents in discharge planning

Department of Health Neonatal Toolkit documents^4^ and NICE guidance^5^ emphasize family‐centred neonatal care, but there is considerable variation in UK NNU family‐centred care, which affects family NNU experiences.^6^ Evidence of NNU family‐centred care practice is limited despite the importance of staff‐mother interactions in facilitating mothers as main caregivers.^7^, ^8^

Transition from hospital to home involves a complex process of adaptation by parents and multidisciplinary approaches to families.^9^ Key elements include discharge planning and the way discharge and adjustment to home takes place, especially when vulnerable babies have been very sick.

### 1.2 Parental confidence

Parents in NNUs focus on discharge, and the care and skills needed to facilitate this. Stress and anxiety reduce their ability to absorb and retain complex changing messages from neonatal staff. Early practical involvement in baby care increases parental confidence and competence, and willingness to take responsibility for care.^3^

North American work on early educational interventions for parents in NNUs indicates enhanced parent‐infant interactions and length‐of‐stay reductions.^10^ Parents’ concerns change in moving from hospital to home and include uncertainty about parenting competence, breast‐feeding proficiency and losing care from neonatal staff.^2^, ^11^, ^12^ Involving parents in discharge planning develops confidence in parenting abilities at home.^9^ Timely discharge information and early anticipatory guidance helps build confidence and role efficacy as discharge approaches.^2^

### 1.3 Developing parent‐oriented approaches to infant discharge planning from NNUs

Parental self‐efficacy and competence are moderated by parent knowledge of development and parenting stress.^13^, ^14^ Maternal efficacy beliefs mediate the effects of depression, social support and infant temperament on parenting behaviours.^15^ Self‐efficacy tools based on Bandura's Social Learning Theory can indicate the level of belief and confidence people have about their ability to plan and carry out specific tasks^16^, ^17^, ^18^

McMaster NNU, Canada, developed an interactive discharge planning tool to achieve timely transfers between various levels of neonatal care and encourage parental engagement.^19^ From this, we developed a UK neonatal planning package (Train‐to‐Home) to improve parents’ preparedness and confidence for discharge. Train‐to‐Home is parent centred and aims to facilitate communication between staff and parents throughout their stay, encouraging early parental involvement in care and developing their understanding of their baby's needs, and provides a realistic estimated baby discharge date soon after admission. It comprises a laminated baby's train graphic for the baby's cot and gestational age appropriate parent booklets. Train‐to‐Home was introduced in four local neonatal units (LNUs) across South West England in 2013. A previous paper reports the wider study findings;^20^ we present here qualitative data comparing parental and neonatal staff perceptions and experiences of using Train‐to‐Home.

## 2 Methods

The main study recruited infants without major anomalies born at 27‐33 weeks’ gestation over two 11 month periods before and after introducing Train‐to‐Home. LNU nurses sought parental assent to take part and consent was gained by a study researcher.

### 2.1 Parent interviews

Parents who agreed to further contact were telephoned two months after discharge and invited to participate in a semi‐structured telephone interview at a time of their choice. We noted the proportions of infant gestational ages, maternal ages and parity in each LNU by phase recruited to the main study and purposively sampled similar proportions for the interviews to reflect the main sample characteristics and achieve a maximum variation sample. Participants varying by ethnicity, socioeconomic status, age and parity, with babies of different gestational ages, and from singleton and multiple births were interviewed.

An interview topic guide was developed with the project Parent Advisory Group, using literature and insights from study management team meetings. Some parents were interviewed before Train‐to‐Home was introduced (Phase 1) and others after its introduction (Phase 2). The guide covered practical and emotional readiness for discharge and staff roles. Attitudes and experiences of Train‐to‐Home were also explored in Phase 2.

Parent interviews were audio‐recorded and transcribed verbatim. NVivo (QSR International Pty Ltd, Victoria, Australia 2010) was used to support thematic analysis using constant comparative techniques to test emerging themes in subsequent interviews.^21^ To ensure validity of the analysis, two researchers read and re‐read the transcripts and interview notes, coding and identifying themes, with two other researchers independently checking and agreeing the themes identified on a subsample of the data. Descriptive accounts were produced, and theoretical explanations for behaviours, opinions and decisions were developed.

### 2.2 Nursing and medical staff focus groups/interviews

After family recruitment was completed, nursing staff focus groups were arranged in each LNU to explore Train‐to‐Home use. Timings were agreed with managers to fit staff rotas. One‐to‐one interviews were arranged for staff unable to attend the groups. A senior researcher conducted short telephone interviews with consultant neonatologists from each LNU. Staff focus groups/interviews were similarly audio‐recorded, transcribed and analysed thematically. Similar checks on coding and identification of emerging theme ensured analysis validity and enhanced rigour.

The descriptive accounts and themes were compared using a framework approach in reviewing parents’, nurses’ and doctors’ perspectives.^22^, ^23^ The themes generated were presented, discussed and endorsed by the study Parent Advisory Group members.

Research ethics approval was given by the NRES Committee London—City & East in June 2012: 12/LO/0944.

## 3 Results

Altogether 245 families participated in the main study (Phase 1—128 families, Phase 2—117). Despite a lack of measurable change in parental self‐efficacy scores at discharge with the intervention, post‐discharge emergency department (ED) visits fell with a significant reduction in associated post‐discharge health‐care costs.

### 3.1 Interview participants

Thirty‐seven telephone interviews lasting 30‐60 minutes were conducted with parents (Phase 1—16, Phase 2—21). Fathers participated in three interviews where both parents contributed to discussions. Babies’ gestational ages at birth spanned 27‐33 weeks. Some parents had twins, and both first time and subsequent parents were represented from all LNUs. Mothers’ median age was 32 years and half had male children.

Eighteen nursing staff (two managers, seven sisters, four staff nurses, two health‐care assistants and three nursery nurses) were interviewed face‐to‐face across the LNUs in groups of three or four, plus a few one‐to‐one. Telephone interviews were held with five neonatal consultants from the four LNUs. The focus groups and interviews captured staff views about Train‐to‐Home materials and how they were used. Quotations are attributed to LNUs to preserve anonymity. Interviews and group duration varied from 30‐60 minutes.

### 3.2 Preparation for discharge home (Phase 1)

Overarching themes arising from parents’, nursing staff and doctors’ interviews are summarized in Table 1, grouped as “practical preparation,” “emotional preparation” and “role of feeding.” The illustrative quotes are from Phase 1 parents.

**Table 1:** Themes arising from preparation for going home from local neonatal unit (LNU)

Preparation for going home from neonatal care	Parents: n=37	Nurses: n=18	Doctors: n=5
Practical preparation	Knowledge and skills transfer, but not enough notice	Gaining weight and maintaining temperature.	Early planning for discharge. Communication with parents
Emotional preparation	Uncertainty, feeling rushed, motivation to get home		
Role of Feeding	Breastfeeding is the harder way to do it	Getting the feeding right	

Parents described their LNU experience as a roller‐coaster emotional journey, where they learnt a great deal, established relationships with nurses and received support. Overall, they felt prepared practically for discharge, but expressed concerns about not having a discharge date or discussions about discharge. There was emotional uncertainty about discharge, and some wanted more reassurance. Breastfeeding was often experienced as difficult and getting it right could delay discharge. Nursing staff focused on babies maintaining their temperature, gaining weight and feeding well before they could be discharged. Doctors concentrated on the need for early discharge planning.

#### 3.2.1 Practical preparation: “knowledge and skills transfer, but not enough notice”

Nurses worked with parents in supporting readiness for baby discharge. Parents said the knowledge gained prepared them practically and most recalled the practical skills they had learnt. Systematic information delivery using checklists or parent packs was not mentioned and the processes seemed reactive not proactive. Mothers expressed concern about lack of discharge notice, not having a date or structured, meaningful discharge discussions. They had difficulty alerting family members about imminent departure and making family arrangements.

It was all very quick in the end. … we didn't really talk about going home and then all of a sudden it was, “Let's try demand feeding. You could go home in two days”..very, very sudden.

(Mother #719)

#### 3.2.2 Emotional preparation: “uncertainty, feeling rushed, motivation to get home”

Mothers described simultaneously feeling “scared,” “nervous” and “excited” about discharge. Some felt unsupported and needed more reassurance. Fear and concern about taking home a “tiny baby” was raised by a small number of mothers, but this was overcome by desire to have the family at home.

It came to the point where we, yeah, we were getting prepared to do it and, yeah, nervous, scared, but excited at the same time…mentally kind of preparing how we were going to do things, continuing it at home rather than having the hospital around to help.

(Mother #521)

#### 3.2.3 Role of feeding: “breastfeeding is the harder way to do it”

Nurses across all LNUs supported and encouraged breastfeeding, giving advice about expressing breast milk and feeding position, but there was conflicting advice. Nurses helped mothers experiencing breastfeeding issues and mothers switching to formula feeding so that their baby could go home sooner. Breastfeeding was seen to be difficult for this group of babies, but it was not clear how nurse‐parent conversations contributed or improved this.

Got loads of support with breastfeeding. You just felt able to, you know, ask questions. They were particularly useful, very supportive, yeah.

(Mother #305)

So that we could go home they said, “Why don't you just do top‐ups with formula? Because then we know that she's got a means of having enough milk when you go home.”

(Mother #514)

Everybody acknowledged that breastfeeding is the harder way of doing it. And a lot of babies that were bottle‐fed left sooner than us.

(Mother #305)

#### 3.2.4 Preparing parents to take their baby home—“gaining weight, feeding and maintaining temperature”

Nursing staff perceived parents’ main issues were babies gaining weight, feeding well (particularly if breastfed) and maintaining their temperature. They spoke about parents worrying about coping and fearing discharge. They talked about using due date as a guide and advising parents that 37 weeks of gestation is more realistic.

We used to say that babies go home about their due date and anything before is a bonus. Now we tend to say…it will probably be between 37 and 38 weeks in most cases. The focus here would be to get them to establish breastfeeding.

(Manager, unit 1)

There is a perceived feeling that if they bottle feed they will get home quicker. For a Mum who is breastfeeding they get frustrated with that last hurdle and they will switch to bottle if most of the others around them are bottle feeding.

(Health‐care assistant unit 1)

All the consultants took a more top‐down approach and focused on the need to find better ways of planning for discharge and communicating this to parents.

There is real value in the process of thinking clearly about discharge planning; we need to do more work on improving information exchange and supporting parents.

(Doctor, Unit 1)

### 3.3 Preparation for discharge home: using Train‐to‐Home (Phase 2)

**Figure 1:**
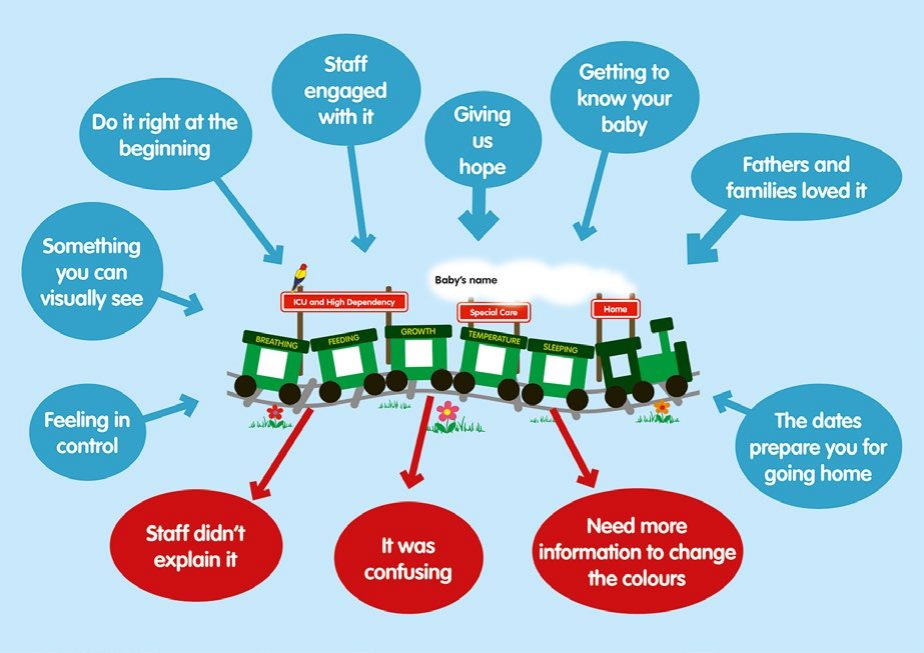
Themes generated from parents’ responses about Train‐to‐Home in Phase 2. Red, yellow or green stickers were put in the train windows as appropriate to indicate preparedness for discharge home, and a discharge date range was added beneath the track [Colour figure can be viewed at wileyonlinelibrary.com]

Train‐to‐Home was introduced, and the baby's train was displayed on the cot with coloured stickers (red‐yellow‐green) indicating the stage of preparedness for discharge home (see Figure 1).^20^ Designed to be kept up‐to‐date by parents in discussion with staff, it aimed to improve parental confidence via engagement and education. Gestational age appropriate booklets encouraged parents to question staff about their baby's care and development.

Parents were overwhelmingly positive about Train‐to‐Home. Mothers, fathers and siblings found the visual representation of baby progress helpful and enjoyable. Negative or critical comments focused on nursing staff attitudes, confused messages and misunderstanding or non‐engagement with the package (Figure 1).

Nursing and medical staff agreed that the intervention materials’ visual nature helped in explaining baby progress to parents, but nursing staff views varied. Doctors reported improved communication with parents, and some nurses said it allowed them to sit and talk with parents about how their baby might progress and baby's developmental changes and needs.

Using a similar framework (Table 2), we compared views about who should use Train‐to‐Home, when it should be used, people's reactions and its content/function.

**Table 2:** Themes arising from the use of Train‐to‐Home

Train‐to‐Home discharge package	Parents: N=21	Nurses: N=18	Doctors: N=5
Who should use it?—parents, staff, others	*The value of staff engagement* *Fathers and families loved it*.	*Discussion with the parents*	*Early planning for discharge*
When should it be given?	*Do it right at the beginning* *The dates prepare you for home*	*Uncertainty about timing*	*Hostility about dates*. *Improving communication with parents*
How does it make us feel? emotional preparation	*Giving us hope* *Communication with parents*	*Giving discharge dates is difficult*	*Giving bad news*
What is included, what it does—practical preparation	*Something you can visually see* *Give me the information to change the colours* *Getting to know your baby*	*Visual images explain progress*.	*The power of visual images*. *Explaining preterm development*

#### 3.3.1 Who should use it?—parents, staff and others

Parent themes illustrate their view that staff needed to be engaged with the process, but fathers and families could also be involved.

##### The value of staff engagement

Parents responded positively where staff engaged and delivered care using Train‐to‐Home, as it helped them understand what was happening.

It was one of the nursery nurses sat down with me and explained what it [Train‐to‐Home] was and did the first round of stickers and then another nurse went through it again a bit later, updated it with a different discharge date and everything.

(Mother #219)

We loved it [Train‐to‐Home], we felt like it made us feel like a part of it. We loved getting the little booklet out, and we would discuss it between the two of us and with the nurses and take pictures of it.

(Mother #617)

Parents had difficulty engaging with Train‐to‐Home if it was not introduced or fully explained. Some were confused by nursing staff comments about the study materials, including criteria for changing train window colours; challenging discharge dates; and using “due date” as the discharge date. In half the LNUs, nursing staff told parents to use their due date as the discharge target and then added a date to the train.

I think nearly all of them [nurses] said that, it [date range] was unrealistic. It seemed very strange, because when it was first presented to us we were kind of like ‘oh wow that's really soon’, … the first thing they said was you have to go with his due date….So that was very confusing.

(Mother #225)

The train appeared one day with some dates on it and… I wasn't around when the nurse came to do it, and then I didn't know what it meant for quite some time. I think it was another couple of weeks before it was explained to me.

(Mother #213)

##### Fathers and families loved it

Mothers identified viewing the train, reviewing criteria, changing stickers and taking photographs to send to their family as helpful activities for fathers and other children.

One of the nurses sat down and went through everything so that we completely understood it, and it was great because we've got a four year old,… he loved the train, he thought it was fantastic, and it was great for him to be able to understand a bit more…

(Mother #418)

It made us feel good when he got an amber [sticker]. I took pictures of the train and showed it to friends, to the grandparents and colleagues at work. Towards the end I was able to say that he'd got four greens and we were just working on the feeding. I thought it made it visual—it was really excellent.

(Father #816)

#### Early planning for discharge

Train‐to‐Home dates were usually changed after discussions with medical staff. Nurses in three LNUs struggled to explain the dates to parents, but nurses in the fourth LNU used the changing dates and window colours to talk with parents about how their baby was progressing. More guidance on changing dates was suggested and some nurses felt this was not their role. Others relied on shared discussion, emphasizing the tentativeness of initial dates and flexibility of response to events that might change discharge timing.

We change the dates in discussion with the doctors. Sometimes the frame is just a little bit too narrow. For me it's an excuse to sit down with the parents and discuss the train and feeding plan.

(Neonatal sister, unit 4)

I think it's been lovely to be able to give them an idea right from the word go. I really liked that, because that is the one thing that they always ask is, “When will my baby be going home?”

(Manager, unit 4)

For medical staff, the intervention provided a focal point from which to start discharge planning starting with LNU admission, but they highlighted difficulties in embedding Train‐to‐Home.

The idea was very good, it “normalised” the process of going home from early in the hospital stay.

(Doctor Unit 3)

There was real value in the process of thinking clearly about discharge planning; we need to do more work on improving information exchange and supporting parents. ….It was very difficult to get nurses to “buy in” to the process—they thought it was “too simplistic.”

(Doctor Unit 1)

#### 3.3.2 When should it be introduced?

##### “The dates prepare you for going home” so “Do it right at the beginning”

Parents talked about the usefulness of preparing for discharge by having a date to work towards.

It just says the date you will be going home, it's just a rough idea about how you can be prepared for it.

(Mother #622)

However, some parents had not received Train‐to‐Home at the beginning of their LNU stay, and this impeded their chance to engage with their baby's progression.

We did think it was a really good idea that you can see him progressing, but it's just unfortunate that we didn't do it right at the beginning…..So we didn't actually see much progress. That's one thing I suggest…is to do it right at the beginning so that you can see a change.

(Mother #408)

#### Hostility about the dates

Some staff on three LNUs thought the intervention should be used in lower dependency areas rather than intensive care on admission. They considered this a more appropriate place, bearing in mind baby needs and staff workload. However, the fourth LNU used Train‐to‐Home from admission and continued to use it beyond the study.

I think there's more time in low dependency. You've got also more of an idea of when that baby is likely to go home, parents relax more about it don't they? They start enjoying their baby rather than being worried about it. Once the baby went to low dependency, we can start thinking about discharge.

(Health Care Assistant, Unit 2)

I think overall it's worked really well, it's been quite positive for the parents, which is the main thing really. We just do it every day now, it's part of the daily thing you do with the parents.

(Neonatal sister, unit 4)

Doctors talked about the difficulty in embedding the intervention in current practice and culture and nurses’ responses, in contrast to the usefulness of using it with parents.

When one or two babies took longer to be ready for home than our initial estimate the nurses became very hostile about the estimates—said they were “too optimistic”

(Doctor, unit 1)

The process did however unmask some “chain of command” problems—some nurses felt very negative about it and we were “too democratic” about how we implemented this.

(Doctor, unit 3)

#### Improving communication with parents

A positive view about early introduction of Train‐to‐Home was evident among medical staff.

This was a very helpful approach that improved communication between us (medical staff) and parents about the planning for sending the baby home. The information for parents was very helpful and parents really appreciated being involved in this from an early stage.

(Doctor, unit 2)

Many parents appreciated being told when to expect their baby to be discharged at the time of admission with a specific “timeline” assigned it. The actual dates of discharge made it easier for parents to prepare at home for the arrival of their baby and staff to appreciate the anticipated duration of each individual patient's admission. Setting a timeline gave parents and staff some sort of “working time schedule”. This greatly enhanced communication between healthcare practitioners and parents.

(Doctor, unit 4)

#### 3.3.3 Emotional preparation—How does it make us feel?

##### “Giving us hope” and “Feeling in control”

Using Train‐to‐Home, changing train stickers from red, through yellow to green gave parents the sense of moving forwards and being on a real journey home. This included feeling hopeful and more in control. In an environment marked by their lack of choice and control, this apparently simple intervention improved things.

The train was one of the only things I remember from those first couple of days… I was in shock, pain, emotional and very tired…I remember the train, remember thinking—OK so he works his way along until he leaves.

(Mother #802)

The train is meant to give you like a bit of hope for when your baby is coming home. We all knew it's not definite, but it's meant to give you that bit of hope.

(Mother #808)

The doctors came round while I was there early and said that I could do the stickers myself. Something so small actually makes a big impact, you kind of feel quite good about being able to put a different colour sticker in, because you can see how she is improving.

(Mother #807)

##### Giving discharge dates is difficult

The difficulty of breaking bad news and wanting to protect parents from disappointment came through in nurses’ comments, and this was reinforced by doctors’ responses.

When they don't reach those dates or you change the dates then they get quite disappointed.

(Neonatal sister Unit 1)

So once you start writing the date on there then the parents are like glued to that date, and it's a bit difficult to then try and move it.

(Staff nurse Unit 2)

Nurses did not like having to give parents the “bad news” that their baby would be in hospital longer than the initial estimated date.

(Doctor Unit 1)

#### 3.3.4 Practical preparation—What is included and what does it do?

Some parents took more ownership of “Train‐to‐Home” and associated discharge planning processes and explained it to their wider families. The traffic light system of colours provided an “at‐a‐glance” affirmation that their baby's health was improving. However, a few reported that nursing staff handed over train maintenance responsibility, but did not give them enough information to use it correctly. Consequently, stickers were not updated as parents did not understand the basis on which to change them.

##### Something you can visually see

It's something you can visually see, because looking at your child in an incubator and the only difference you can really see is maybe a few tubes are coming out, but when you can look at the colours obviously going from red to green and that it's all positive.

(Mother #807)

I really liked it, I thought it was a great idea, because it's a quick easy way of seeing where they are. When my parents came, instead of having to explain everything I could show them the little train, with their colours on there.

(Mother #219)

I think it just needs a little bit more work around explaining it to parents, who is leading on it really, and what's the criteria so that you can make judgements about where your baby is.

(Mother #621)

##### Getting to know your baby

Many parents used the booklet and some added comments or observations. They enjoyed using it and found the questions helpful. Mothers and fathers found it facilitated engagement and understanding, helping them to ask questions.

It kind of gave us a guide to what we should be doing and what we should be learning and looking for.

(Mother #617)

I did find it useful, like when we looked for the questions, and going through each stage with the nurses….especially the questions in there that you probably didn't think of yourself. So it was good to look in there and we felt that we could ask them.

(Mother #408)

#### Explaining preterm development

Nursing staff views about using Train‐to‐Home were mixed. They used it to explain baby progression as it helped start discussions; however, some were ambivalent. In contrast, nursing staff in the fourth LNU embraced Train‐to‐Home and appreciated the underlying principles. Doctors talked about the benefits of the train image and of the additional information presented concerning the key complicated physiological concepts underpinning preterm babies’ health and development.

Seeing that visual representation that their baby is getting better, that they are making that journey towards home. It's fantastic; it's been a really good way of engaging the parents with their baby's progress. It's definitely a good starting point for communication with them

(Manager, unit 4)

Parents felt more empowered in the care of their baby and this tool made it easier for them to understand the clinical course of their baby's admission. It makes it easier for parents to understand the concept of a baby being “physiologically ready for discharge” ie what physiological maturation that must occur before their baby can go home.

(Doctor, Unit 4)

Nurses were aware that parents used the pathway booklets to help them map out events and anticipate possible directions and engaged with the information provided.

It's interesting looking at the moment at some of the developmental work that's being done and I think the whole pathway idea working with parents is a really positive one.

(Manager, unit 1)

They feel they're able to ask. It's almost like they need the verification of the book to say to you, “In the book it says… so does that mean that I can? Some of them used them daily and read questions out, or underlined things, and wrote how their babies were doing, like a diary really.

(Nursery nurse, unit 4)

## 4 Discussion

Parents interviewed before introducing the intervention expressed concerns about the uncertainties associated with hospital discharge. After Train‐to‐Home was introduced, interviewees were overwhelmingly positive. They described being given hope, feeling in control and having something visual to show their baby's progress. They spoke about fathers’ and families’ involvement, how predicted discharge dates helped preparation for home and the ways staff engaged with the materials when communicating with them. Staff reactions were mixed—one LNU was very positive, and in other LNUs, more junior nurses and special care staff were more ready to engage with the principles. Medical staff in all four LNUs were positive about the intervention as it improved communication with parents.

Nursing staff mentioned the NHS discharge planning initiative^4^ and placing babies in their gestational developmental journey. Train‐to‐Home fitted well with these approaches.

Similar findings are presented in the Canadian study using a Train‐to‐Home approach. Some staff saw the discharge planning pathway as a barrier because of its perceived redundancy.^19^ A web‐based family care map supporting NNU family‐based care practices was developed in New England, US.^24^ This tool was used to help individual care providers and family advisors provide comprehensive family‐centred care to infants and families, with the potential to affect the quality of newborn intensive care positively and lead to improved long‐term outcomes.^24^ Our Train‐to‐Home materials are now similarly available as web‐based resources to enable staff and parents to access the parent pathways and train.

Despite the rhetoric about family‐centred NNU care and planning for discharge,^1^, ^4^, ^5^ these processes remain reactive and poorly planned.^6^, ^7^ A study in NICUs in England showed that mothers did not report positive family‐centred practices.^7^ Mothers experienced role uncertainty or ambiguity and were acutely sensitive to care inconsistencies.^7^ To minimize this, staff‐parent interactions must be improved and mothers’ and fathers’ opportunities to be main caregivers facilitated.^7^ Our study implemented a family‐centred care package, and where staff adopted it, changes in parental confidence in caring for their baby were seen when they left the NNU and particularly in the following weeks.^20^

Maternal self‐efficacy studies have explored the associations with later maternal behaviour.^15^, ^25^ Our study measured maternal self‐efficacy, and parents reported feeling more confident and better informed about caring for their babies after Train‐to‐Home was introduced.^20^ They very clearly welcomed partnership working with professionals and needed realistic information about a likely discharge date. Shared discussions of problems and questions that might arise at each stage of their baby's pathway through the NNU were positive and helpful.

Attempts to change NNU communication practices should involve parents as change agents as they will engage positively and enthusiastically contribute their experiences even at times of extreme emotional stress and rapid change. Although fathers were much less involved with data collection in this study than mothers, they and the wider family are important and should not be ignored.

### 4.1 Study strengths and limitations

Participating families showed high levels of engagement and commitment to the main study and the qualitative component, despite having very young preterm infants and being under considerable stress. Interviews were representative of the population who took part in the wider study.^20^ Recruitment of families from more‐deprived groups was lower than from less‐deprived groups in common with studies involving families from wide socioeconomic backgrounds.

More time was probably needed to embed and normalize Train‐to‐Home into each LNU as some staff were uncertain about using the package. Medical staff supported Train‐to‐Home's underpinning principles; however, they identified some nursing staff's reluctance to engage fully and were therefore unwilling to promote the intervention to unenthusiastic nurses.

## 5 Conclusions

Using a parent‐centred approach to communication and informing parents about the needs and progress of their preterm infant in hospital is both practical and welcomed by parents and many staff. This approach meets the recommended prioritization of family‐centred care for such families. The predicted discharge dates helped parents prepare for home and the ways that staff engaged with Train‐to‐Home when communicating with them helped them feel more confident, as well as having something visual to show their baby's progress.

## Conflict of Interests

None.
